# The upstream regulatory mechanism of BplMYB46 and the function of upstream regulatory factors that mediate resistance to stress in *Betula platyphylla*

**DOI:** 10.3389/fpls.2022.1030459

**Published:** 2022-10-25

**Authors:** Huiyan Guo, Xiaomeng Sun, Bo Wang, Di Wu, Hu Sun, Yucheng Wang

**Affiliations:** ^1^ College of Forestry, Shenyang Agricultural University, Shenyang, Liaoning, China; ^2^ The Key Laboratory of Forest Tree Genetics, Breeding and Cultivation of Liaoning Province, Shenyang Agricultural University, Shenyang, Liaoning, China

**Keywords:** BplMYB46, *Betula platyphylla*, upstream regulatory factors, *cis*-*acting* elements, stress resistance

## Abstract

Previously, we have shown that the transcription factor BplMYB46 in *Betula platyphylla* can enhance tolerance to salt and osmotic stress and promote secondary cell wall deposition, and we characterized its downstream regulatory mechanism. However, its upstream regulatory mechanism remains unclear. Here, the promoter activity and upstream regulatory factors of BplMYB46 were studied. Analyses of β-glucuronidase (GUS) staining and activity indicated that *BplMYB46* promoter was specific temporal and spatial expression, and its expression can be induced by salt and osmotic stress. We identified three upstream regulatory factors of BplMYB46: BpDof1, BpWRKY3, and BpbZIP3. Yeast-one hybrid assays, GUS activity, chromatin immunoprecipitation, and quantitative real-time polymerase chain reaction revealed that BpDof1, BpWRKY3, and BpbZIP3 can directly regulate the expression of *BplMYB46* by specifically binding to Dof, W-box, and ABRE elements in the *BplMYB46* promoter, respectively. BpDof1, BpWRKY3, and BpbZIP3 were all localized to the nucleus, and their expressions can be induced by stress. Overexpression of *BpDof1*, *BpWRKY3*, and *BpbZIP3* conferred the resistance of transgenic birch plants to salt and osmotic stress. Our findings provide new insights into the upstream regulatory mechanism of BplMYB46 and reveal new upstream regulatory genes that mediate resistance to adverse environments. The genes identified in our study provide novel targets for the breeding of forest tree species.

## Introduction

Plants are sessile organisms that are continually exposed to different types of abiotic and biotic stress, and they possess various mechanisms to cope with adverse environmental conditions and resist attack by other organisms (Hickman et al., 2013; Chen et al., 2019; Iqbal et al., 2021). Several genes are involved in the regulation of stress responses. Transcription factors (TFs) can bind to specific *cis*-*acting* elements on promoters to control the expression of various target genes; they thus play important roles in the growth and development of plants (Franco-Zorrilla and Solano, 2017; Wang et al., 2021a).

The MYB family is one of the largest families of TFs in plants; MYB TFs play key roles in primary and secondary metabolism and regulate resistance to biotic and abiotic stresses and other physiological processes in plants (Mitra et al., 2019; Berardi et al., 2021; Xiao et al., 2021; Cao et al., 2022). Over expression of *MYB6* in transgenic poplar up-regulates the expression of flavonoid biosynthesis genes and significantly increases the accumulation of anthocyanins and proanthocyanidins in *Populus tomentosa* (Wang et al., 2019). MdMYB308L positively regulates the accumulation of anthocyanins and cold tolerance in apple (An et al., 2020). BpMYB4 promotes stem development and cellulose biosynthesis and regulates tolerance to abiotic stress in birch (Yu et al., 2021). Overexpression of *MbMYB108* from *Malus baccata* enhances resistance to cold and drought stress in transgenic *Arabidopsis thaliana* plants (Yao et al., 2022). Some studies have shown that MYB TFs can regulate the expression of target genes and affect the response to various signals in plants (Xue et al., 2019; Wang et al., 2022b). For example, PavMYB10.1 in sweet cherry regulates the expression of *PavANS* and *PavUFGT* by binding to the AE-box, a light response element in their promoters, and promotes anthocyanin synthesis (Jin et al., 2016). Yeast-one hybrid (Y1H) assays and electrophoretic mobility shift assays (EMSA) have shown that CiMYB42 regulates the expression of *CiOSC* by binding to the MYB core element in its promoter to positively regulate limonoid biosynthesis in citrus (Zhang et al., 2020). MYB21 plays a key role in regulating flavonol biosynthesis by directly binding to the GARE *cis*-*acting* element in the *FLS1* promoter in *A. thaliana* (Zhang et al., 2021). MdMYB2 directly binds to the MBS motif in the SUMO E3 ligase *MdSIZ1* promoter and activates the expression of *MdSIZ1*, which affects cold tolerance and the accumulation of anthocyanins in apple (Jiang et al., 2022). PnMYB2 in *Panax notoginseng* binds to the promoters of *PnCesA3* and *PnCCoAOMT1* to regulate their expression; these genes promote primary cell wall (PCW) and secondary cell wall (SCW) biosynthesis by enhancing cellulose and lignin biosynthesis (Shi et al., 2022). Although the downstream regulatory mechanisms of MYB TFs have been well studied, the upstream regulatory mechanisms of MYB TFs have been less well studied by comparison. The upstream regulators of TFs mediate the responses to various signals. Given that upstream regulators of TFs have global effects in transcriptional regulation, additional studies are needed to clarify the upstream regulatory mechanisms of TFs.

Previously, we have shown that BplMYB46 in *Betula platyphylla* (birch), a pioneer species of secondary forests in northeastern China, enhances tolerance to salt and osmotic stress and promotes SCW deposition; we have also characterized its downstream regulatory mechanism (Guo et al., 2017; Guo et al., 2018). However, its upstream regulatory mechanism remains unclear. Here, the promoter activity and upstream regulatory factors of BplMYB46 were analyzed. We also studied the functions of the upstream regulatory factors of BplMYB46. The results of our study provide new insights into the upstream regulatory mechanism of BplMYB46. Our findings will also aid the discovery of novel upstream regulatory genes that promote resistance to adverse environments and provide targets for the breeding of new forest tree species.

## Materials and methods

### Plant materials

The stems and leaves of birch were cut into small pieces and incubated on woody plant medium (WPM + 0.5 mg/L 6-BA + 0.5 mg/L KT). After the calli regenerated, they were transferred to growth medium (WPM + 1.0 mg/L 6-BA) for bud differentiation. Adventitious buds with 3–5-cm shoots were cut and transferred to root generation medium (WPM + 0.2 mg/L NAA) in a growth chamber with a 12 h/12 h light/dark cycle, an average temperature of 26°C, and 70–75% relative humidity.

Birch seeds were cultivated on Woody Plant Medium (WPM with 2.5% (w/v) sucrose and 0.6% (w/v) agar (pH 5.8) in a growth chamber and then planted in pots containing a mixture of perlite/vermiculite/soil (1:1:3) in a greenhouse. Conditions in the growth chamber were as follows: 12 h/12 h light/dark cycle, average temperature of 26°C, and 70–75% relative humidity. The plants were thoroughly watered every day.

### Plasmid construct and plant transformation

Genomic DNA was extracted from *B. platyphylla* using the CTAB method (Cheng et al., 2003). A 1,443-bp sequence of the *BplMYB46* promoter excluding 5’ untranslated region (UTR) was amplified and then cloned into the pCAMBIA1301 vector to replace the cauliflower mosaic virus (CaMV) 35S promoter driving the beta-glucuronidase (*GUS*) gene. The primers used are listed in **Table S1**. The *BplMYB46* promoter::*GUS* construct was electroporated into Agrobacterium competent cells (EHA105) and then transformed into *B. platyphylla* using the *Agrobacterium tumefaciens*-mediated transient transformation method well applied in herbaceous and woody plants (Zang et al., 2017; Hu et al., 2019), according to a previous protocol (Ji et al., 2014) with some modifications. Briefly, single colony of *A. tumefaciens* strain EHA105 harboring *BplMYB46* promoter::*GUS* was grown in Luria-Bertani (LB) liquid medium (containing 50 mg/L kanamycin and 50 mg/L rifampicin) at 28°C with shaking. After overnight incubation, 1ml of culture was transferred to 50 ml of fresh LB liquid media and incubated at 28°C with shaking. Cells were harvested by centrifugation at 3,000 rpm for 10 min when the culture density reached an OD_600_ of 0.5–0.6, and were resuspended in the transformation solution (1/2 MS + sucrose [2.0%, w/v] + 10 mM CaCl_2_ + 120 μM acetosyringone + 200 mg/L DTT + Tween-20 [0.02%, v/v], pH 5.8] as the transformation solution for the transformation study. For transient genetic transformation, the birch plants at different developmental stages were soaked in the transformation solution and shaken at 120 rpm and 25°C in the dark. After 24 h, the birch samples were collected.

### GUS staining and activity analysis

The birch plants of BplMYB46 promoter::*GUS* transient genetic transformation, including 5-day-old seedlings, 10-day-old seedlings, 14-day-old seedlings, 30-day-old seedlings, and mature leaves of 45-day-old seedlings were collected. Next, β-glucuronidase (GUS) staining was performed according to previous method (Zheng et al., 2012). The *BplMYB46* promoter::*GUS* transient transgenic birch plants for one-month-old were watered with 150 mM NaCl or 200 mM mannitol solution for 6, 12, and 24 h. Specifically, taking 24 h as the final treatment time point, 6 h and 12 h treatments were carried out backwards. The control plants were watered for 24 h with water. All birch plants were collected at the 24th h, then were homogenized in GUS extraction buffer. The supernatant was assayed for GUS activity with 4-methylumbellifery-β-d-glucuronic acid as the substrate; fluorescence values were measured using 4-methylumbelliferon as the calibration control. The activity of GUS was determined using previously published method (Gampala et al., 2001). Data were shown as the mean of three biological replicates.

### Identification of the upstream regulatory factors of BplMYB46 using Y1H assays

The total RNA of one-month-old birch plants was extracted. The cDNA library was obtained *via* the Matchmaker^®^ Gold Yeast One-Hybrid Library Construction & Screening Kit (Clontech) and used as the effector construct. The *BplMYB46* promoter was inserted into multiple cloning sites of the pHIS2 plasmid (*EcoR I* and *Sac I*) to drive *HIS3* expression and used as the reporter construct. The primers used are shown in **Table S2**.

The cDNA library (effector) and *BplMYB46* promoter (reporter) were co-transformed into Y187 yeast cells using the Y1H technique. The Y1H system consisted of the following: 3 µg of *Sma I*-linearized pGADT7-Rec2, 2–5 µg of cDNA library, 5 µg of reporter construct, and 20 µL of carrier DNA. The mixture was added to 600 μL of competent Y187 yeast cells. The transformation was performed using the Yeast Transformation System 2 (Clontech). The Y187 construct was grown on SD/-Trp/-His/(DDO) and SD/-Trp/-His/-Leu/(TDO), and TDO contained 50 mM 3-AT (3-amino-1, 2, 4-triazole) medium. The yeast plasmids were extracted from monoclonal colonies cultured on TDO medium with 50 mM 3-AT using the Easy Yeast Plasmid Isolation Kit (Clontech); they were then transformed into Escherichia coli DH5α competent cells through heat shock and cultured on LB medium containing ampicillin to identify the TFs that bind to the promoter sequence of *BplMYB46*. Positive clones were detected *via* PCR (primers are shown in **Table S3**) and were sequenced.

### Y1H verification

The truncated *BplMYB46* promoter with and without Dof, W-box, and ABRE *cis*-*acting* elements; the three tandem copies of DNA sequences of the Dof, W-box, and ABRE *cis*-*acting* elements; the three tandem copies of mutated DNA sequences (A/T was mutated to C, G/C was mutated to A) of the Dof, W-box, and ABRE were cloned into the pHIS2 vector and used as reporters. Primers used and sizes of amplicons are shown in **Table S4**. The pGADT7-Rec2-*BpDof1*, pGADT7-Rec2-*BpWRKY3*, and pGADT7-Rec2-*BpbZIP3* constructs were used as effectors. The effectors and their corresponding reporters were co-transformed into yeast cells (Y187) and selected on TDO medium containing 50 mM 3-AT. The positive control was the interaction between pGADT7-Rec2-p53 and pHIS2-p53. The negative control was the interaction between pGADT7-Rec2-p53 and pHIS2-*BplMYB46* promoter.

### ChIP analysis

The open reading frames (ORFs) of *BpDof1*, *BpWRKY3*, and *BpbZIP3*, without the termination codon, were separately inserted into the vector pBI121 upstream of green fluorescent protein (GFP) under the control of the CaMV 35S promoter. The pBI121-35S::*BpDof1*-*GFP*, pBI121-35S::*BpWRKY3*-*GFP* and pBI121-35S::*BpbZIP3*-*GFP* construct was transformed into *Agrobacterium* EHA105 competent cells and then into the one-month-old birch plants by *A. tumefaciens*-mediated transient transformation, respectively. To determine whether the upstream regulators could bind to specific *cis-acting* elements of the *BplMYB46* promoter to regulate its expression *in vivo*, ChIP analysis was conducted following a previously described method (Zhao et al., 2020) with some modifications. Briefly, transgenic birch plants (1–5 g) transiently expressing *BplDof1*, *BpWRKY3*, and *BpbZIP3* were collected and crosslinked with 3% formaldehyde for 10 min at room temperature in a vacuum. The cross-linking was quenched by 2 mol/L glycine for 2 min in a vacuum at room temperature, followed by five washes with deionized water. Tissue was then ground into a fine powder using liquid nitrogen. The purified cross-linked nuclei were sonicated to shear the chromatin into 0.2–0.8 kb fragments, and 1/10 volume was saved as the input control. One portion of chromatin was immunoprecipitated with GFP antibody (ChIP+). The other portion was immunoprecipitated without antibody as a negative control (ChIP−). The immunoprecipitated complexes were incubated at 65°C for 12 h to release the DNA fragments. The immunoprecipitated DNA was extracted with chloroform for purification. The DNA fragments containing or lacking Dof, W-box, and ABRE elements in the *BplMYB46* promoter region were selected for amplification. The thermal cycling conditions for PCR were as follows: 95°C for 5 min; 35 cycles of 94°C for 30 s, 56°C for 30 s, and 72°C for 30 s; and a final incubation at 72°C for 7 min. Enrichment of truncated promoters in the immunoprecipitated samples was determined by quantitative PCR (qPCR). The thermal cycling protocol was as follows: 95°C for 30 s; 40 cycles at 95°C for 10 s, 55°C for 10 s, and 72°C for 30 s; and 60°C for 15 s for plate reading. The tubulin gene was used as an internal control. Three biological replicates were conducted. All the primers used are shown in **Table S5**.

### Validation by transient expression assay

The binding ability of upstream regulatory factor to the specific element in *BplMYB46* promoter was validated using the *GUS* reporter gene assay. The truncated promoter including or lacking the Dof, W-box or ABRE elements was fused with the CaMV 35S minimal promoter (46 bp to +1, replaced 35S promoter) to drive the *GUS* gene. *BpDof1*, *BpWRKY3*, and *BpbZIP3* was inserted into the pROKII vector under the control of the CaMV 35S promoter and used as effector, respectively. Each effector of pROKII-35S::*BpDof1*, pROKII-35S::*BpWRKY3* and pROKII-35S::*BpbZIP3* was co-transformed with each reporter into one-month-old birch plants by *Agrobacterium tumefaciens*-mediated transient expression. The GUS activity was determined. Data are represented as the mean of three biological replicates. The primers are listed in **Tables S6**.

### Expression analysis of BplMYB46

To determine whether the upstream regulatory factors can regulate the expression of *BplMYB46*, each effector of pROKII-35S::*BpDof1*, pROKII-35S::*BpWRKY3* and pROKII-35S::*BpbZIP3* was transformed into the one-month-old birch plants using the transient transformation method. The RNA was extracted using a Universal Plant RNA Extraction Kit (BioTeke Corporation, China). The cDNA was synthesized from approximately 1 μg of total RNA using PrimeScript IV First-Strand cDNA Synthesis Mix (TaKaRa, Japan). Quantitative real-time PCR (qRT-PCR) of *BplMYB46* was conducted with 10 μL of SYBR Green Real-time PCR Master Mix (BioTake Corporation, China), 1 μL of cDNA template, 1 μL of forward primer (10 μM), and 1 µL of reverse primer (10 μM); the final reaction volume was adjusted to 20 µL with ultrapure water. The thermal cycling conditions were as follows: 94°C for 30 s; 94°C for 12 s, 58°C for 30 s, and 72°C for 45 s for 45 cycles; and 79°C for 1 s for plate reading using a qTOWER^3^ G system (Analytik Jena AG, Germany). After the final PCR cycle, the temperature was increased from 55°C to 99°C at 0.5°C per s to generate the melting curve for the samples. Three independent experiments were performed. The *tubulin* (GenBank accession number: FG067376) and *ubiquitin* (GenBank accession number: FG065618) genes were used as internal controls. All primers and amplicon sizes are shown in **Table S7**. The expression of *BplMYB46* for each sample was calculated using the delta-delta CT method (Pfaffl et al., 2002).

### Subcellular localization analysis of upstream regulatory factors

The pBI121-gene-GFPs and pBI121-GFP (control) were separately transformed into onion epidermal cells using particle bombardment (BioRad). After incubation on 1/2MS medium for 24 h in the dark, the transformed onion epidermal cells were stained with DAPI (100 ng/mL) and visualized under a confocal laser-scanning microscope (A1, Nikon, Japan).

### qRT-PCR analysis of upstream regulatory factors

After approximately 2 months of cultivation, healthy birch seedlings approximately 25 cm in height with similar growth conditions were treated with 200 mM NaCl or 300 mM mannitol for 0.5, 12, 24, and 48 h. Specifically, taking 48 h as the final treatment time point, 0.5 h, 12 h and 24 h treatments were carried out backwards. The control plants were treated with fresh water for 48 h. The birch seedlings were collected at the 48th h. Three independent biological replicates were conducted, and each replicate comprised six seedlings. All samples were quickly frozen using liquid nitrogen and stored at –80°C.

Total RNA was extracted and treated with DNase I; it was then reverse-transcribed into cDNA using the PrimeScript™ RT reagent Kit (Takara). qRT-PCR was performed for *BpDof1*, *BpWRKY3*, and *BpbZIP3*. The primers and amplicon sizes of the genes are shown in **Table S8**. All the procedures and parameters for qRT-PCR were the same as those described above.

### Stress tolerance analyses of plants overexpressing upstream regulatory factors

The pROKII-35S::gene and pROKII-35S empty vector were separately transformed into one-month-old birch seedlings using the transient transformation method. The transient transgenic plants were treated with 150 mM NaCl or 200 mM mannitol for 12 h. Control plants were treated with water. The detached leaves of birch plants were incubated with 0.5 mg/mL nitroblue tetrazolium (NBT, dissolved in phosphate buffer, pH 7.8) and 0.5 mg/mL 3’-diaminobenzidine (DAB, dissolved in phosphate buffer, pH 3.8) as described in a previous study (Zhang et al., 2011). Evans blue (1.0 mg/mL, dissolved in sterile deionized water) staining was conducted to detect cell death following previously published procedures (Kim et al., 2003). The activity of superoxide dismutase (SOD) and peroxidase (POD), the content of H_2_O_2_, and electrolyte leakage were measured following previously described methods (Liu et al., 2015; Wang et al., 2015). The concentration of protein in plants was detected using a kit produced by Nanjing Jiancheng Bioengineering Institute. Three independent biological replicates were conducted.

### Statistical analysis

All statistical analyses were conducted in SPSS software (IBM, IL, USA), and analysis of variance was used to evaluate the significance of differences between groups. The threshold for statistical significance was *P <*0.05.

## Results

### The temporal and spatial expression of *BplMYB46* promoter

**Figure 1:**
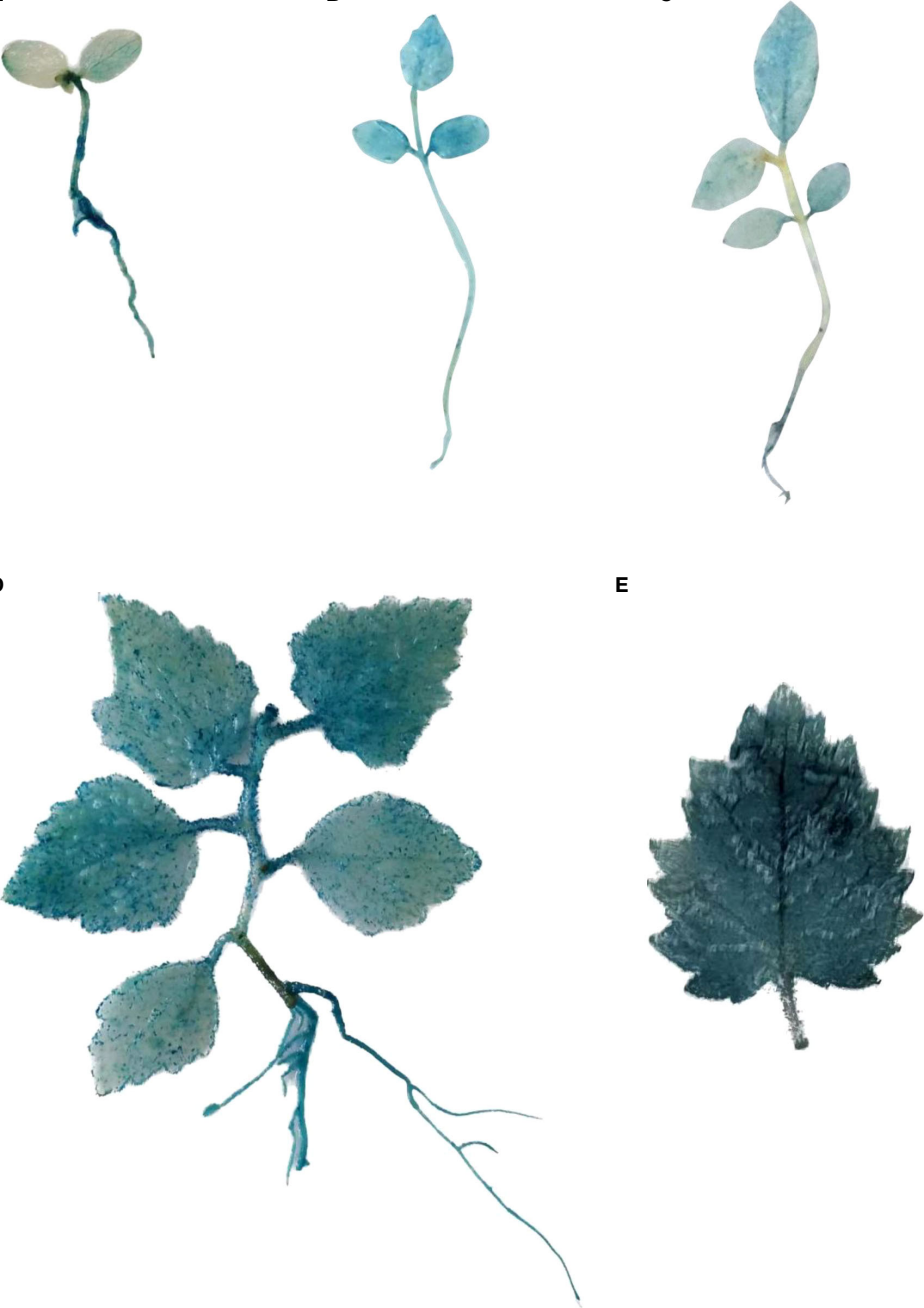
The temporal and spatial expression of *BplMYB46* promoter in transient transgenic *BplMYB46* promoter::*GUS* birch plants. The *BplMYB46* promoter::*GUS* construct was transformed into birch plants and β-glucuronidase (GUS) histochemical staining was performed to investigate the temporal and spatial expression of *BplMYB46* promoter. **(A)** 5-day-old seedling; **(B)** 10-day-old seedling; **(C)** 14-day-old seedling; **(D)** 30-day-old seedling; **(E)** mature leaf of 45-day-old seedling.

The *BplMYB46* promoter::*GUS* construct was transiently transformed into birch plants using *A. tumefaciens*-mediated transformation. The expression pattern of *BplMYB46* promoter in birch was performed *via* GUS staining (**Figure 1**). GUS activity was detected at every developmental stage and in almost all tissues of birch plants. At the initial developmental stage of birch seedlings, GUS activity of the hypocotyls was higher than that of the cotyledons (**Figure 1A**). Interestingly, GUS activity of the leaves and roots was higher than that of the stems, with the continuous development of birch seedlings (**Figures 1B, C**). Moreover, GUS activity was much higher in old leaves than in young leaves (**Figures 1D, E**), and GUS activity of the leaf veins was higher than the rest of the leaf (**Figure 1E**). Our findings suggest that *BplMYB46* promoter have the temporal and spatial expression specificity.

### *BplMYB46* promoter activity under abiotic stress

**Figure 2:**
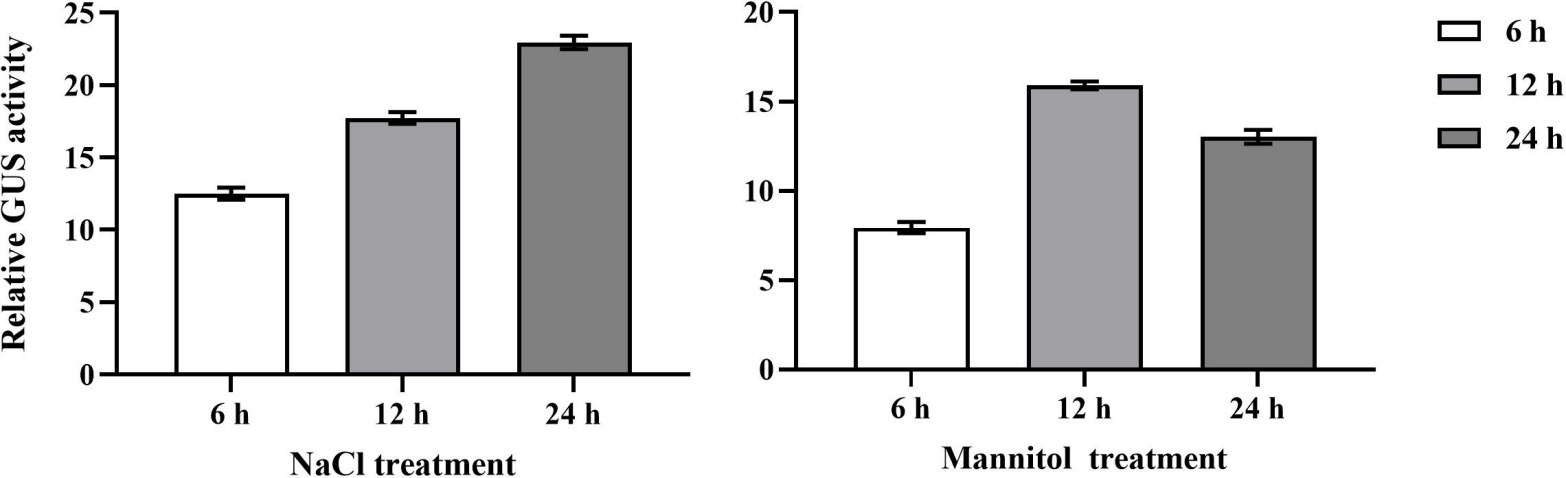
Relative activity of *GUS* in transient transgenic *BplMYB46* promoter::*GUS* birch plants under salt and osmotic stress. Relative activity of GUS under salt stress of 150 mM NaCl and osmotic stress of 200 mM mannitol for 6 h, 12h and 24 h. *BplMYB46* promoter::*GUS* construct was transformed into one-month-old birch plants using the *Agrobacterium tumefaciens*-mediated transient transformation method. The relative activity of *GUS* mean that the relative level of *GUS* in transient transgenic *BplMYB46* promoter::*GUS* birch plants under salt and osmotic stress, compared with that in transient transgenic *BplMYB46 promoter*::*GUS* birch plants under water treatment. Error bars indicate the standard deviation of three biological replicates.

To clarify the roles of the *BplMYB46* promoter in response to stress, transient transgenic *BplMYB46* promoter::GUS birch plants were exposed to salt and osmotic stress for different lengths of time, and the relative GUS activity was determined (**Figure 2**). Our results showed that GUS activity was up-regulated under salt and osmotic stress compared with the control. Specifically, GUS activity increased from 6 h to 24 h under salt treatment; under osmotic treatment, GUS activity first increased from 6 h to 12 h and then decreased from 12 h to 24 h. Our findings suggested that the *BplMYB46* promoter can respond to abiotic stress and that its expression can be induced by salt and osmotic treatment *in vivo*.

### Analysis of the *cis*-*acting* elements of *BplMYB46* promoter

**Figure 3:**
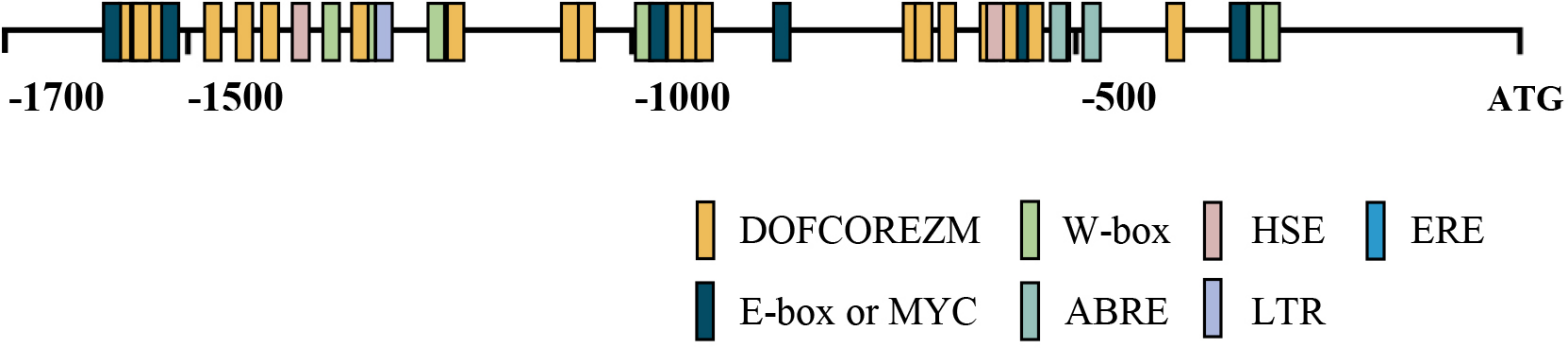
Distribution of *cis*-*acting* elements in *BplMYB46* promoter. The 7 different *cis*-*acting* elements in the promoter of *BplMYB46* gene are represented in different color boxes.

The *cisacting* elements in the promoter sequence of *BplMYB46* excluding 5’ UTR were predicted using the PlantCARE database. Some *cisacting* elements in *BplMYB46* promoter were identified (**Figure 3**), including DOFCOREZM, E-box/MYC, W-box, ABRE, HSE, LTR, and ERE elements, which are involved in abiotic stress, light, abscisic acid, and ethylene responsiveness. In them, DOFCOREZM, E-box/MYC, W-box and ABRE were the more abundant *cisacting* elements than the rest elements (**Table 1**).

**Table 1:** Predicted *cis*-*acting* elements in the *BplMYB46* promoter.

Name	Core sequence	Number of copies
DOFCOREZM	AAAG	25
E-box or MYC	CANNTG	11
W-box	TGAC	6
ABRE	ACGTG	4
HSE	AAAATT	2
LTR	CCGAAA	1
ERE	ATTTCAAA	1

### Identification of the upstream regulatory factors of BplMYB46

**Figure 4:**
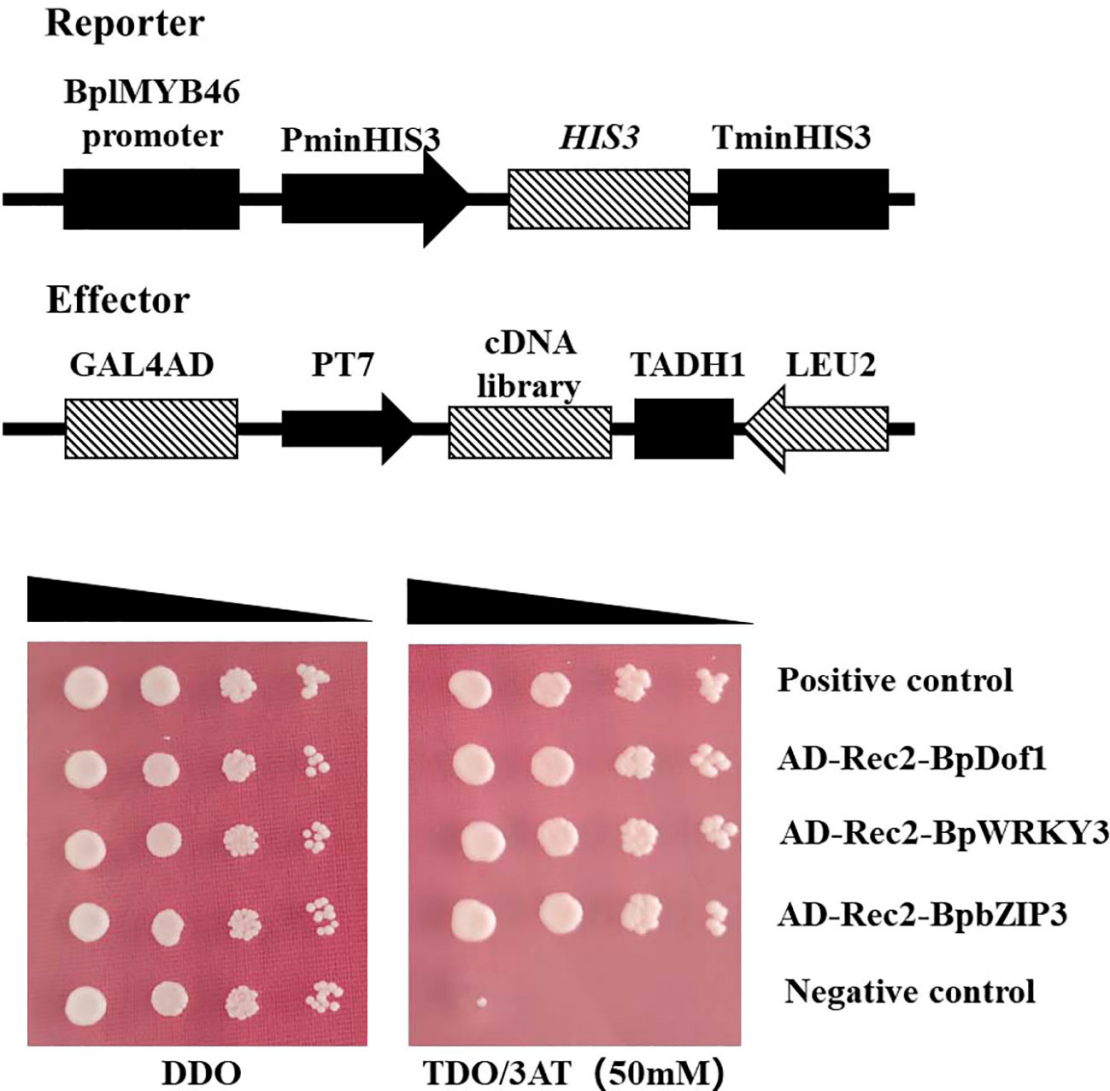
Three regulatory factors that bound to the *BplMYB46* promoter were identified from the birch cDNA library *via* Y1H analysis. **(A)** Schematic diagram of the effector and reporter used in the Y1H analysis. **(B)** Verification of the birch cDNA library (effector) and the *BplMYB46* promoter (reporter) constructs co-transformed into yeast Y187 cells. The positive transformants were determined by spotting the serial dilutions (1:1, 1:10, 1:100, and 1:1000) of yeast onto DDO and TDO plates with 3-AT. Positive control: pGADT7-p53/pHIS2-p53; Negative control: pGADT7-p53/pHIS2-*BplMYB46* promoter.

To identify upstream regulatory factors, the *BplMYB46* promoter was cloned into the pHIS2 vector, which was used as a reporter, and co-transformed into Y187 yeast cells with the cDNA library of birch plants for Y1H analysis (**Figure 4A**). The yeast plasmids were extracted from monoclonal colonies cultured on TDO medium containing 50 mM 3-AT and then were transformed into *E. coli* DH5α. The positive clones were screened on LB medium with ampicillin and then were sequenced. We identified a total of three upstream regulatory factors, BpDof1 (GenBank number: MT075779), BpWRKY3 (GenBank number: OP265743), and BpbZIP3 (GenBank number: OP265744), according to the BLAST sequence analysis tool on the NCBI website (**Figure 4B**).

### Analysis of the interaction between regulatory factors and specific elements

**Figure 5:**
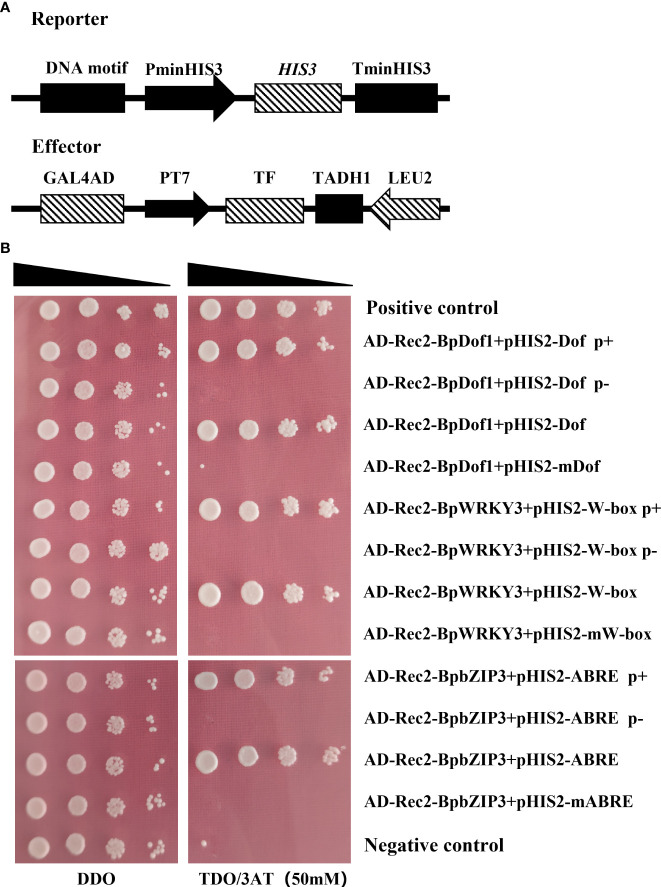
Analysis of the upstream regulatory factors that bind to the Dof, W-box, and ABRE elements by Y1H assays. **(A)** Schematic diagrams of the reporter and effector vectors. **(B)** Verification of the BpDof1, BpWRKY3, and BpbZIP3 effectors and reporter constructs of specific DNA motifs co-transformed into yeast Y187 cells. The DNA motifs are as follows: Dof p+, Dof p-, Dof, and mutated Dof elements; W-box p+, W-box p-, W-box, and mutated W-box elements; ABRE p+, ABRE p-, ABRE, and mutated ABRE elements. Dof p+, W-box p+, and ABRE p+: truncated *BplMYB46* promoter containing Dof, W-box, and ABRE elements, respectively (located at -794 bp – -564 bp, -1347 bp – -1149 bp, and -534 bp – -348 bp upstream of the ORF of *BplMYB46*, respectively). Dof p-, W-box p-, and ABRE p-: truncated *BplMYB46* promoter lacking Dof, W-box, and ABRE elements, respectively (located at -706 bp – -582 bp, -1305 bp – -1149 bp, and -480 bp – -348 bp upstream of the ORF of *BplMYB46*, respectively). Dof, W-box, and ABRE elements: three tandem copies of Dof, W-box, and ABRE elements, respectively. Mutated Dof, W-box, and ABRE elements: three tandem copies of mutated Dof, W-box, and ABRE elements, respectively (A/T was mutated to C, G/C was mutated to A). TF means BpDof1, BpWRKY3, and BpbZIP3 transcription factor, respectively. Positive control: pGADT7-p53/pHIS2-p53; Negative control: pGADT7-p53/pHIS2-*BplMYB46* promoter.

The interactions of BpDof1, BpWRKY3, and BpbZIP3 effectors with the truncated *BplMYB46* promoter with and without Dof, W-box, and ABRE elements; with three tandem copies of Dof, W-box, and ABRE elements; and with their mutated sequences were analyzed using Y1H assays (**Figure 5A**). BpDof1, BpWRKY3 and BpbZIP3 bound to the truncated *BplMYB46* promoter containing Dof, W-box, and ABRE elements and three tandem copies of Dof, W-box, and ABRE elements but failed to bind to the truncated *BplMYB46* promoter lacking Dof, W-box, and ABRE elements and three tandem copies of mutated Dof, W-box, and ABRE elements (**Figure 5B**). Our results further indicated that BpDof1, BpWRKY3, and BpbZIP3 can specifically bind to the Dof, W-box, and ABRE elements, respectively.

### Interaction of upstream regulatory factors with specific elements *in planta*

**Figure 6:**
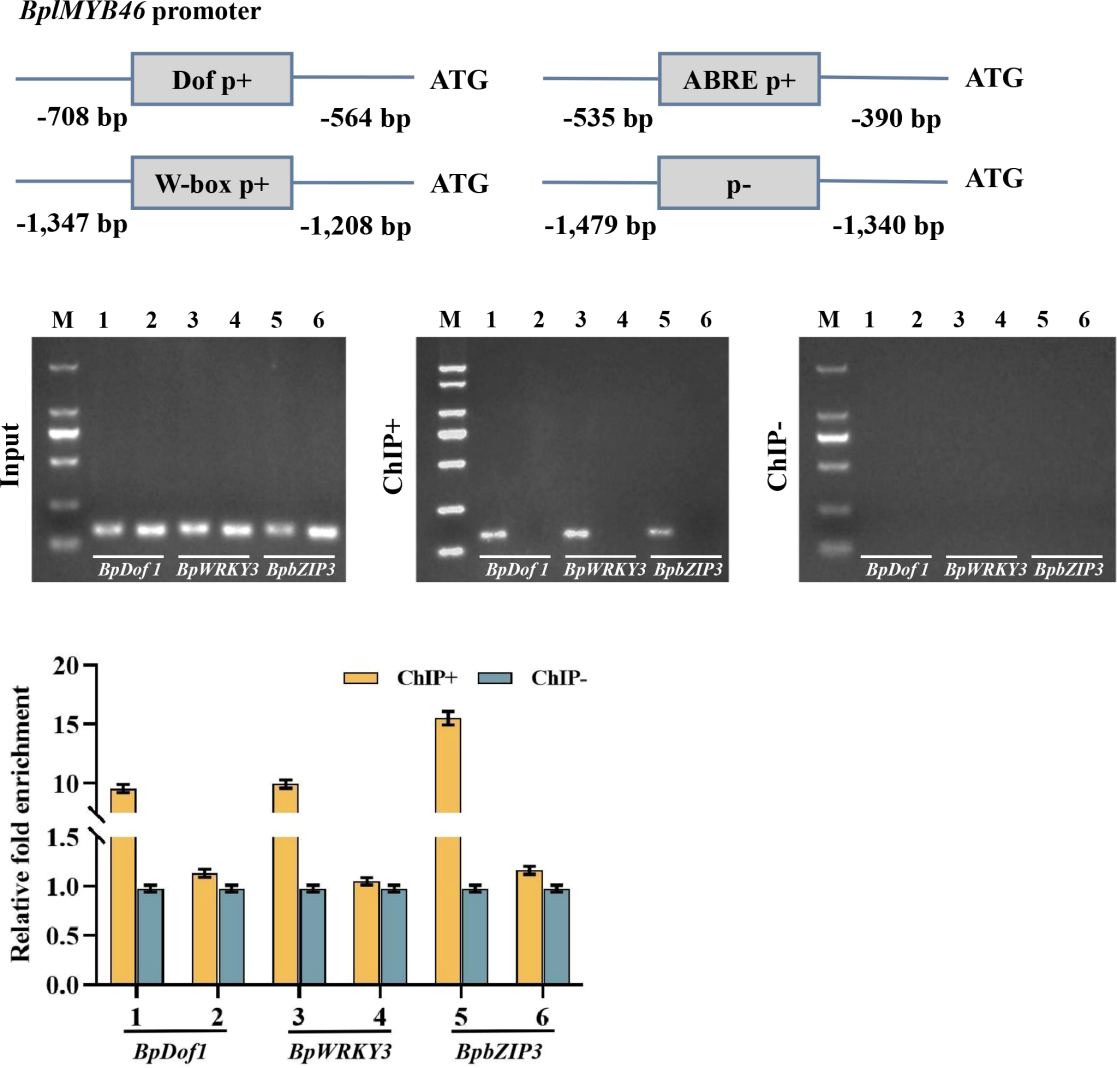
The upstream regulatory factors of BplMYB46 specifically binding to the Dof, W-box, and ABRE elements according to ChIP assays. **(A)** Positions of truncated *BplMYB46* promoters containing and lacking the Dof element. Dof p+, W-box p+, and ABRE p+: truncated *BplMYB46* promoter containing three copies of the Dof element, two copies of the W-box element, and two copies of the ABRE element, respectively. p-: truncated *BplMYB46* promoter without Dof, W-box, and ABRE elements. **(B)** ChIP products obtained from the promoter of *BplMYB46* analyzed by gel electrophoresis after PCR amplification. M: DL2000 Marker (from top to bottom: 2 kb, 1 kb, 750 bp, 500 bp, 250 bp, and 100 bp). **(C)** Real-time quantitative PCR analysis showing the enrichment of the promoter sequence of *BplMYB46* after ChIP. Input, Input DNA (positive control); CHIP+: chromatin immunoprecipitation with anti-GFP antibody; CHIP−: chromatin immunoprecipitation without antibody (negative control). 1: Dof p+, 3: W-box p+, 5: ABRE p+, 2, 4, 6: p-.

The binding between the three upstream regulatory factors and specific *cisacting* elements *in planta* was analyzed using ChIP. The pBI121-35S::*BpDof1*-*GFP*, pBI121-35S::*BpWRKY3*-*GFP* and pBI121-35S::*BpbZIP3*-*GFP* construct was separately transformed into birch plants. *BplMYB46* promoter fragments with Dof, W-box, and ABRE elements (**Figure 6A**) bound by BpDof1, BpWRKY3, and BpbZIP3 TFs were separately captured by ChIP using GFP antibody. ChIP-PCR results revealed that the *BplMYB46* promoter fragments containing the Dof, W-box, and ABRE elements could be enriched by ChIP using GFP antibody (**Figure 6B**). However, the truncated promoter lacking Dof, W-box, and ABRE elements was not enriched by ChIP with GFP antibody (ChIP+) compared with the positive control input and the negative control (ChIP−) (**Figure 6B**). ChIP-qPCR revealed that the promoter region of *BplMYB46*, including the Dof, W-box, and ABRE elements, was significantly enriched in ChIP+, compared with ChIP− (**Figure 6C**). These findings indicated that BpDof1, BpWRKY3, and BpbZIP3 could specifically bind to the Dof, W-box, and ABRE elements *in vivo*, respectively.

### The binding ability of upstream regulatory factors to specific elements

**Figure 7:**
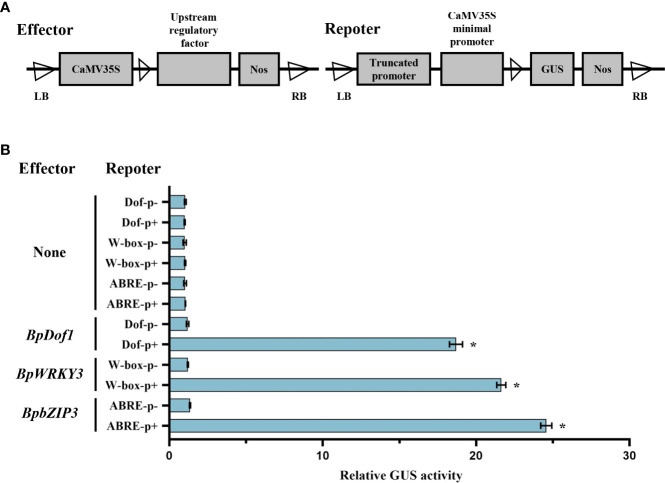
Analysis of upstream regulatory factors binding to the truncated promoters including Dof, W-box, or ABRE elements in birch plants. **(A)** Schematic representation of upstream regulatory factor and the truncated promoter including or lacking the Dof, W-box, or ABRE elements used in the co-expression of effector and reporter in birch plants. The upstream regulatory factor: pROKII-35S::*BpDof1*, pROKII-35S::*BpWRKY3* or pROKII-35S::*BpbZIP3*. Dof p+, W-box p+, and ABRE p+: the truncated *BplMYB46* promoter containing Dof, W-box, and ABRE elements, respectively. Dof p-, W-box p-, and ABRE p-: the truncated *BplMYB46* promoter lacking Dof, W-box, and ABRE elements, respectively. **(B)** Transient co-transformation of effector and reporter constructs in birch plants to study the binding of BpDof1, BpWRKY3 and BpbZIP3 to Dof, W-box, and ABRE elements, respectively. GUS activity indicated the binding affinity of different upstream regulatory factor to the specific truncated promoter. Error bars indicate the standard deviation of three biological replicates. * indicates a significant difference (*P* < 0.05).

To further substantiate the interaction between upstream regulatory factor and the truncated *BplMYB46* promoter containing specific elements, we tested the interaction using the GUS reporter assay. Each effector of pROKII-35S::*BpDof1*, pROKII-35S::*BpWRKY3* and pROKII-35S::*BpbZIP3* was co-transformed with each truncated promoter containing or lacking Dof, W-box, or ABRE elements into birch plants (**Figure 7A**). Only the truncated promoter containing or lacking Dof, W-box, or ABRE elements, without effector, were designated as controls. The results ((**Figure 7B**) indicated that relative GUS activity was much higher in the transformed lines harboring the Dof, W-box, and ABRE than the control, which was about 18, 21 and 24 times that of the control, respectively. So, our results demonstrated that the high binding ability of BpDof1, BpWRKY3 and BpbZIP3 to the Dof, W-box, and ABRE elements in *BplMYB46* promoter, respectively.

### Analysis of *BplMYB46* expression under regulation by upstream regulatory factors

**Figure 8:**
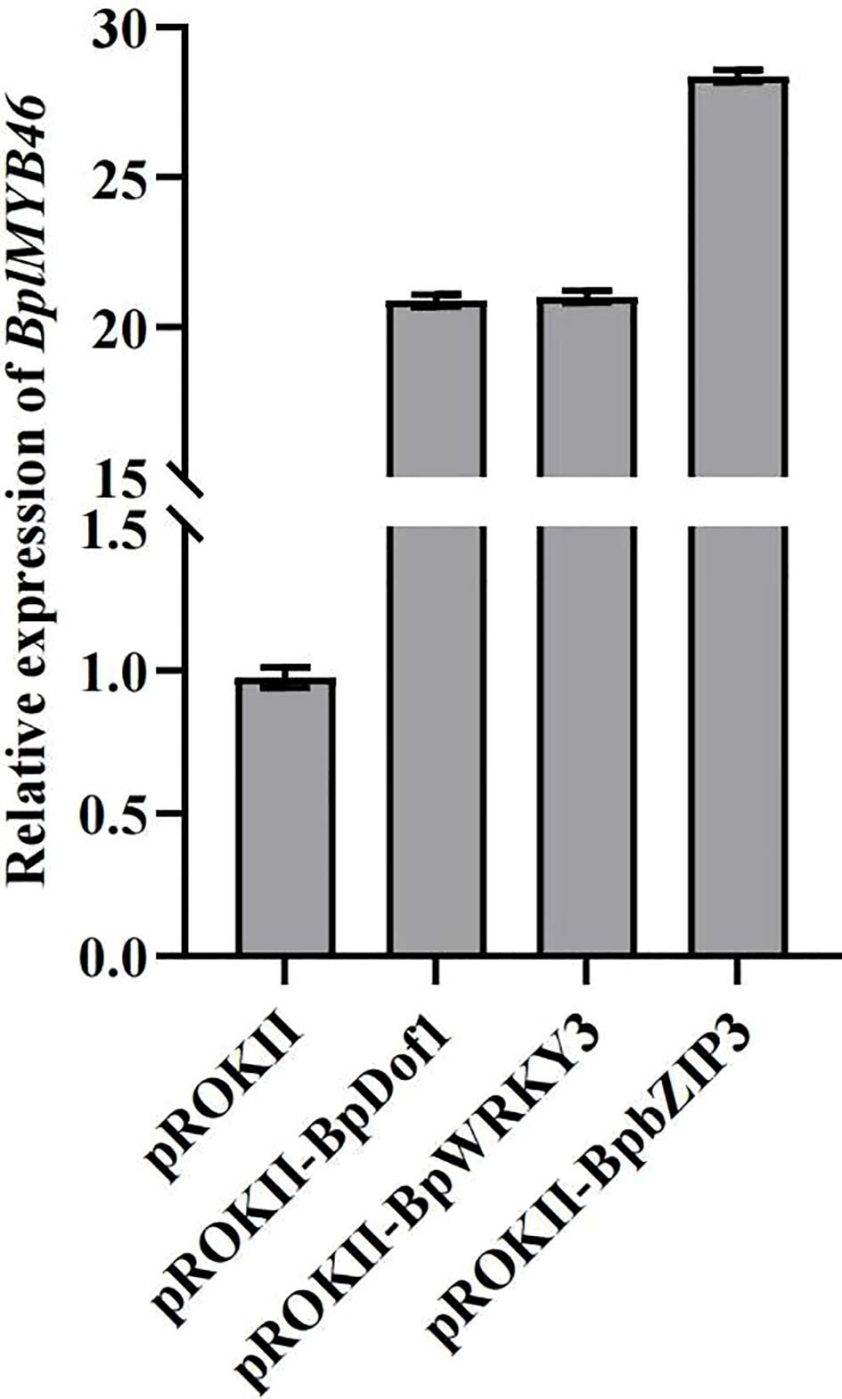
The relative expression levels of *BplMYB46*. The effector, including pROKII-35S:: *BpDof1*, pROKII-35S:: *BpWRKY3* and pROKII-35S:: *BpbZIP3*, was transiently transformed into one-month-old birch plants, respectively. The plants transformed with the empty pROKII vector were used as the control. Error bars indicate the standard deviation of three biological replicates.

To determine whether the upstream regulatory factors can regulate the expression of *BplMYB46*, we transformed each effector of pROKII-35S::*BpDof1*, pROKII-35S::*BpWRKY3* and pROKII-35S::BpbZIP3 into birch using the transient transformation method to generate the overexpression plants, respectively. The relative expression of *BplMYB46* was analyzed using qRT-PCR. The relative expression of *BplMYB46* was much higher in *BpDof1*, *BpWRKY3*, and *BpbZIP3*-transformed birch plants compared with control plants transformed with the empty pROKII vector. The results further indicated that BpDof1, BpWRKY3, and BpbZIP3 can regulate the expression of *BplMYB46* by specifically binding to Dof, W-box, and ABRE elements in *BplMYB46* promoter (**Figure 8**).

### Subcellular localization of the three regulatory factors

**Figure 9:**
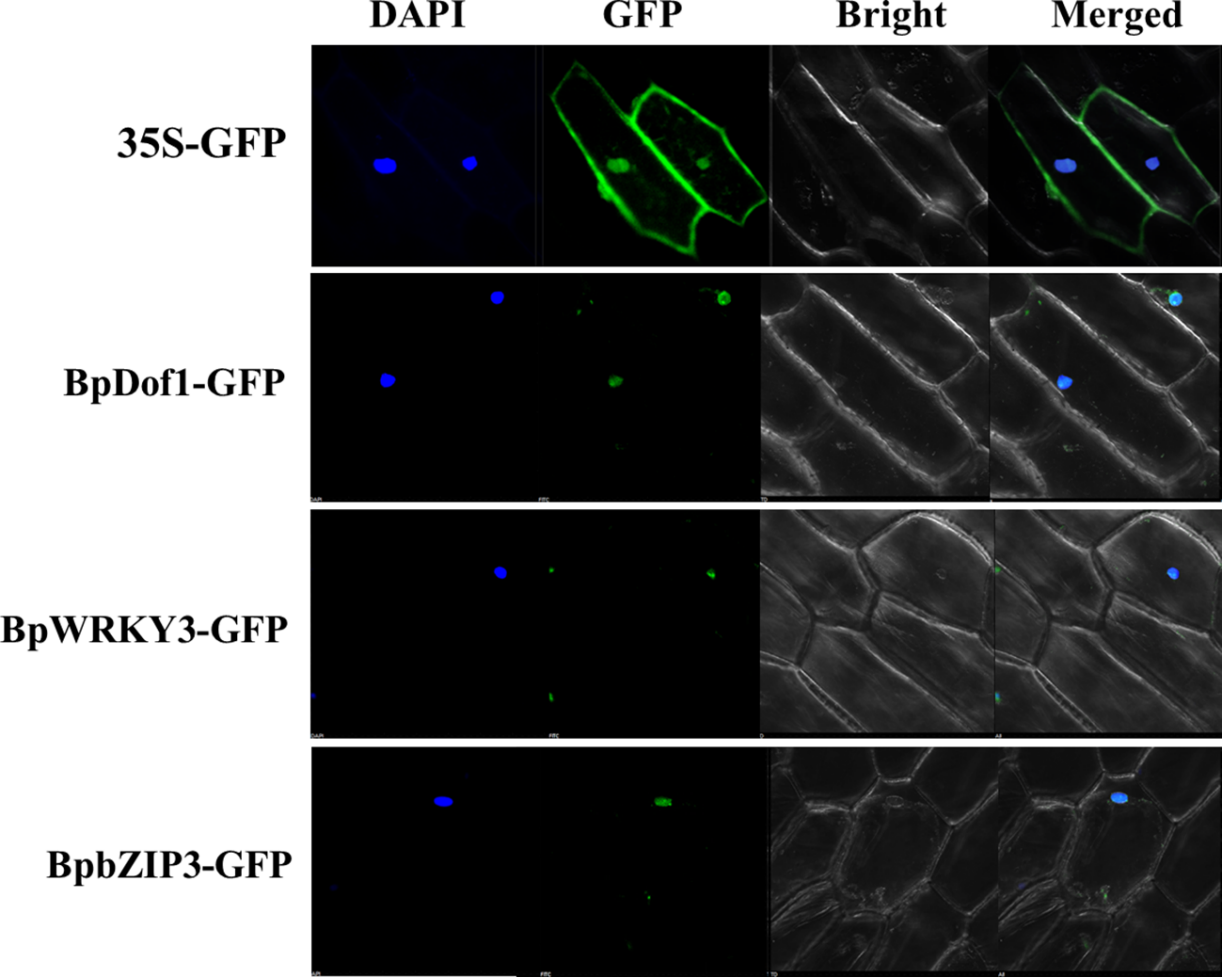
Subcellular localization of three upstream regulatory factors. The fusion genes of *BpDof1*, *BpWRKY3*, and *BpbZIP3* with *GFP*, with 35S-*GFP* as the control, were transiently expressed in onion epidermal cells using the particle bombardment method. The transformed cells were cultured on 1/2 Murashige-Skoog (1/2 MS) medium for 24 h and visualized using a confocal microscope at 488 nm. DAPI: DAPI staining of nuclei; GFP: GFP fluorescence detection; Bright: bright field; Merge: the DAPI, GFP, and bright field images merged.

The fusion genes of regulatory factors with GFP were transformed into onion epidermal cells by particle bombardment using 35S::GFP as the control. The 35S::GFP signals were uniformly distributed throughout the cell, but the green fluorescent signals from BpDof1-GFP, BpWRKY3-GFP, and BpbZIP3-GFP-transformed cells were detected in the nuclei, which were stained using DAPI (**Figure 9**). Our findings indicated that BpDof1, BpWRKY3, and BpbZIP3 are all nuclear proteins similar to BplMYB46.

### Expression patterns of the upstream regulatory factors in response to different types of abiotic stress

**Figure 10:**
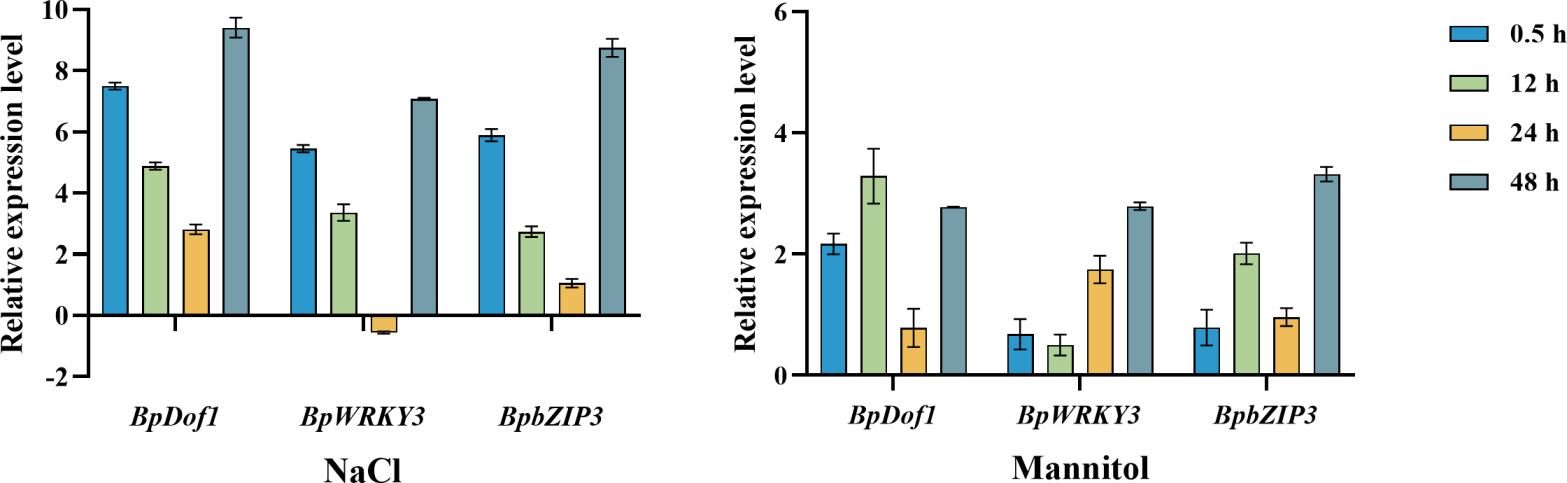
Expression patterns of *BpDof1*, *BpWRKY3*, and *BpbZIP3* under different types of abiotic stress. Two-month-old birch seedlings were treated with 200 mM NaCl and 300 mM mannitol for different lengths of time. Plants watered with fresh water were used as control. After these treatments, birch plants were harvested and pooled for RT-PCR analyses. The error bars indicate the standard deviation of three biological replicates.

To clarify the expression patterns of *BpDof1*, *BpWRKY3*, and *BpbZIP3* in response to salt and osmotic stress, qRT-PCR analyses were conducted (**Figure 10**). Under salt stress, the expression of all three genes was up-regulated from 0.5 h to 48 h relative to the control (water treatment), with the exception of *BpWRKY3* at 24 h, and their expression levels were highest at 48 h. Under osmotic stress, the expression of these three genes was up-regulated from 0.5 h to 48 h relative to the control. The expression levels of *BpWRKY3* and *BpbZIP3* were highest at 48 h, whereas the expression of *BpDof1* was highest at 12 h. Our findings indicated that the expression of *BpDof1*, *BpWRKY3*, and *BpbZIP3* can be induced by salt and osmotic stress in birch plants.

### *BpDof1*, *BpWRKY3*, and *BpbZIP3* overexpression mitigates oxidative stress and cell membrane damage

**Figure 11:**
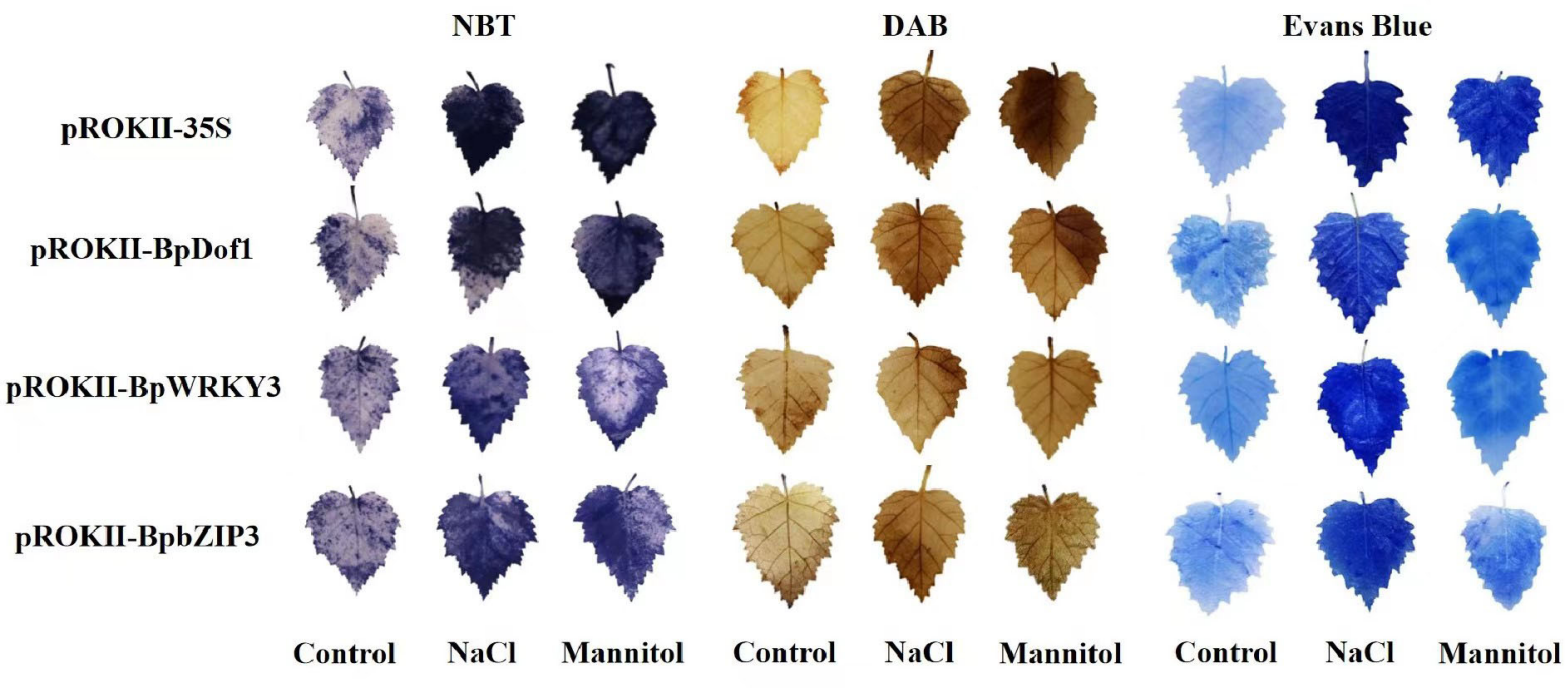
Analysis of ROS accumulation and cell membrane damage in overexpressing *BpDof1*, *BpWRKY3*, and *BpbZIP3* plants under salt and osmotic stress. Birch plants overexpressing *BpDof1*, *BpWRKY3*, and *BpdZIP3* were generated by transient transforming them with pROKII-35S::*BpDof1*, pROKII-35S::*BpWRKY3*, and pROKII-35S::*BpbZIP3*, respectively. The empty pROKII was transiently transformed into birch plants. After exposure to salt stress of 150 mM NaCl and mannitol stress of 200 mM for 12 h. Plants watered with fresh water were used as control. All birch plants were individually stained with NBT to visualize O^2−^ levels and with DAB to visualize H_2_O_2_ levels. Evans blue staining was conducted to visualize cell membrane damage.

To study reactive oxygen species (ROS) accumulation, NBT and DAB *in situ* staining of overexpressing *BpDof1*, *BpWRKY3*, and *BpbZIP3* were performed, which can stain two prominent ROS, O^2−^ and H_2_O_2_, respectively (**Figure 11**). The leaves of *BpDof1*, *BpWRKY3*, *BpbZIP3*, and pROKII-35S plants were stained with NBT and DAB; stained leaves from water-treated plants were used as controls. Under salt and osmotic stress, O^2−^ and H_2_O_2_ levels in the leaves of plants overexpressing *BpDof1*, *BpWRKY3*, and *BpbZIP3* were greatly reduced compared with those in pROKII-35S plants. The content of O^2−^ and H_2_O_2_ indicates the ROS-scavenging ability of plants. Our findings thus indicated that plants overexpressing *BpDof1*, *BpWRKY3*, and *BpbZIP3* had enhanced ROS-scavenging abilities. Evans blue staining was conducted to detect cell membrane damage. The blue staining of plants overexpressing *BpDof1*, *BpWRKY3*, and *BpbZIP3* was less intense compared with that of pROKII-35S plants under salt and osmotic stress, indicating that the extent of cell death was reduced in transgenic plants compared with pROKII-35S plants.

### Physiological characterization of plants overexpressing *BpDof1*, *BpWRKY3*, and *BpbZIP3*

**Figure 12:**
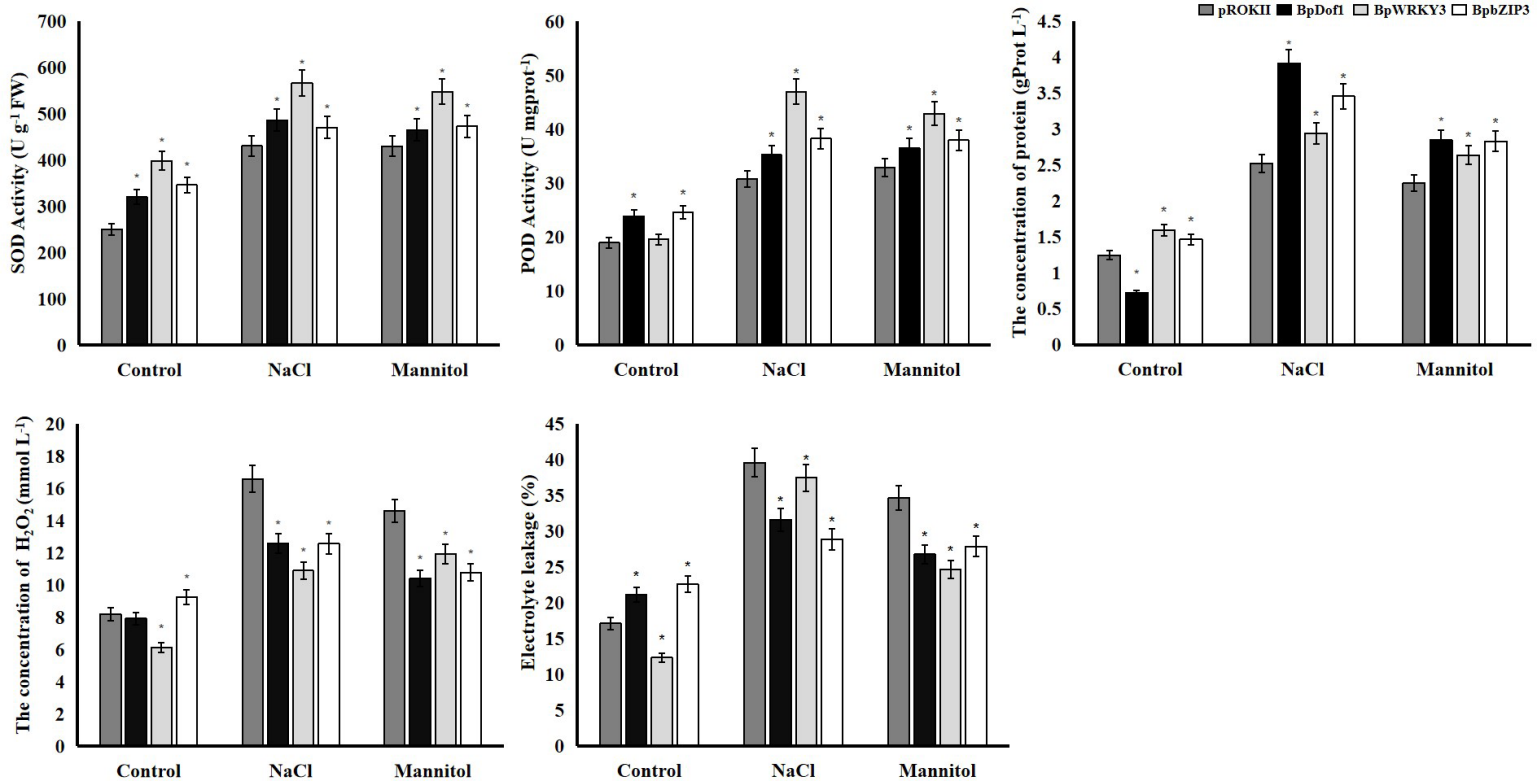
Physiological analyses of overexpressing *BpDof1*, *BpWRKY3*, and *BpbZIP3* plants under salt and osmotic stress. Birch plants overexpressing *BpDof1*, *BpWRKY3*, and *BpdZIP3* were generated by transient transformation method. The empty pROKII was transiently transformed into birch plants. All birch plants were treated using salt of 150 mM NaCl and mannitol of 200 mM for 12 h. Plants watered with fresh water were used as control. All birch plants were individually detected the activity of SOD and POD, content of protein and H_2_O_2_ and electrolyte leakage. The error bars indicate the standard deviation of three biological replicates. * indicates a significant difference (*P* < 0.05).

Activities of superoxide dismutase (SOD) and, peroxidase (POD) activities, the content of soluble protein and H_2_O_2_, and electrolyte leakage are often used to analyze the stress tolerance of plants. We characterized the activity of SOD and POD, the content of soluble protein and H_2_O_2_, and electrolyte leakage to evaluate the resistance of plants overexpressing *BpDof1*, *BpWRKY3*, and *BpbZIP3* and control plants transformed with pROKII-35S to salt and osmotic stress (**Figure 12**). The activity of SOD and POD was higher in plants exposed to salt and osmotic stress than in control plants. The activity of SOD and POD was significantly higher in plants overexpressing *BpDof1*, *BpWRKY3*, and *BpbZIP3* than in pROKII-35S plants, and the activity of SOD and POD was highest in plants overexpressing *BpWRKY3*. The concentrations of protein were higher in plants under abiotic stress compared with control plants. Concentrations of protein were significantly higher in plants overexpressing *BpDof1*, *BpWRKY3*, and *BpbZIP3* than in pROKII-35S plants, and the concentration of protein was highest in plants overexpressing *BpDof1* under salt and osmotic stress. The H_2_O_2_ level was significantly lower in plants overexpressing *BpDof1*, *BpWRKY3*, and *BpbZIP3* than in pROKII-35S plants under salt and osmotic stress. The H_2_O_2_ level was lowest in plants overexpressing *BpWRKY3* under salt stress and in plants overexpressing *BpDof1* under osmotic stress. Electrolyte leakage was lower in the three transgenic plants than in pROKII-35S plants under salt and osmotic stress. Electrolyte leakage was lowest in plants overexpressing *BpDof1* under salt stress and in plants overexpressing *BpWRKY3* under osmotic stress. These findings indicate that *BpDof1*, *BpWRKY3*, and *BpbZIP3* can enhance the ROS-scavenging ability of plants and inhibit cell death.

## Discussion

### Three regulatory factors directly bind to specific *cis*-*acting* elements in the promoter to regulate *BplMYB46* expression

Abiotic stress is a main factor that limit plant growth and development, and TFs play key roles in abiotic stress responses (Xu et al., 2018). Previously, we have shown that BplMYB46 can enhance resistance to salt and osmotic stress in birch plants *via* gain-of-function and loss-of-function analyses (Guo et al., 2017). In the current study, the GUS expression driving by *BplMYB46* promoter was highly induced in birch plants in response to salt and mannitol (**Figure 2**). These results suggest that *BplMYB46* plays an important role in the response to abiotic stress. As BplMYB46 plays a regulatory role, its upstream regulatory factors should play more important regulatory roles, thus, studies of the upstream regulatory factors of BplMYB46 are more meaningful to clarify the molecular mechanism underlying the resistance of birch to stress. Previous reports found that Dof, WRKY and bZIP TFs can bind to Dof, W-box and ABRE elements, respectively (Yanagisawa, 2002; Rushton et al., 2010; Wang et al., 2022a). In the present study, Y1H and ChIP results both showed that three upstream regulatory factors of BplMYB46, named BplDof1, BpWRKY3, and BpbZIP3, can also specifically bind to the Dof, W-box, and ABRE elements in the *BplMYB46* promoter, respectively. TFs regulate the expressions of the target genes *via* binding to the *cis-acting* elements in the promoters. For instance, the TF GmNFYA can regulate the expression of *GmZF392* and *GmZF351* by binding to the CCAAT box in their promoter regions, in soybean (Lu et al., 2021). ThNAC12 can directly regulate the expression of *ThPIP2;5* by binding to the NACRS element in the *ThPIP2;5* promoter (Wang et al., 2021b). In this study, GUS activity analysis further verified the high binding ability of the upstream regulatory factors and the specific *cis-acting* elements (**Figure 7**), and the relative expression of *BplMYB46* was much higher in the overexpressing transient transgenic birch plants of *BplDof1*, *BpWRKY3*, and *BpbZIP3* compared with control plants (**Figure 8**), which indicates that the three genes directly regulate the expression of *BplMYB46* by binding to the Dof, W-box, and ABRE elements in its promoter, respectively.

### The upstream regulatory factors are localized to the nucleus and respond to salt and osmotic stress

In this study, the three upstream regulatory transcription factors of *BplMYB46*, namely BplDof1, BpWRKY3, and BpbZIP3, are all localized to the nucleus (**Figure 9**) like to BplMYB46 (Guo et al., 2017). Previous studies have indicated that most plant TFs are localized to the nucleus, and play their regulatory role (Wang et al., 2015; Wu et al., 2017; Ren et al., 2020), and our results are consistent with these studies. In *Cleistogenes songorica*, the expression of *Dof* genes can respond to high/low temperature, salinity, and ABA treatment (Wang et al., 2021a). In *Spirodela polyrhiza*, the expression patterns of SpWRKYs under phosphate starvation, cold, and submergence treatment indicate that most SpWRKYs are involved in the response to different types of abiotic stress (Zhao et al., 2021). Gene expression patterns and qRT-PCR results indicate that four *JcbZIP*s in Jatropha *curcas* are key stress resistance-related genes under drought and salinity stress (Wang et al., 2021c). Our qRT-PCR results also revealed that three upstream regulatory factors, *BplDof1*, *BpWRKY3*, and *BpbZIP3* can respond to salt and osmotic stress, suggesting they may be involved in stress-signaling pathways.

### *BpDof1*, *BpWRKY3*, and *BpbZIP3* enhance resistance to salt and osmotic stress when they are overexpressed in transgenic birch plants

Plants are often exposed to various types of stress, and this can result in the accumulation of ROS (Wang et al., 2005), and ROS scavenging is thus an important mechanism by which plants resist various types of stress (Zhang et al., 2011). SOD and POD are important antioxidant enzymes and they play the vital roles in ROS scavenging in plants (Wu et al., 2013). Our findings indicate that *BpDof1, BpWRKY3, and BpbZIP3* can enhance the ROS-scavenging ability of plants by increasing the activity of SOD and POD. Some studies have shown that the accumulation of soluble protein in cells plays an osmoregulatory role and thus can increase the resistance to osmotic stress in plants (Parvaiz and Satyawati, 2008; Hanif et al., 2021). Our study showed that overexpression of *BpDof1*, *BpWRKY3*, and *BpbZIP3* can improve stress tolerance by regulating soluble sugar accumulation to balance osmotic pressure. Evans blue staining and electrolyte leakage can affect cell death in plants, as manifested by damage to the cell membrane (Wang et al., 2014). Our results indicated that *BpDof1*, *BpWRKY3*, and *BpbZIP3* can improve stress resistance of plants by reducing the extent of cell death, according to Evans blue staining and electrolyte leakage analysis.

### An upstream regulatory model presenting the function of BplMYB46 in the response to abiotic stress

**Figure 13:**
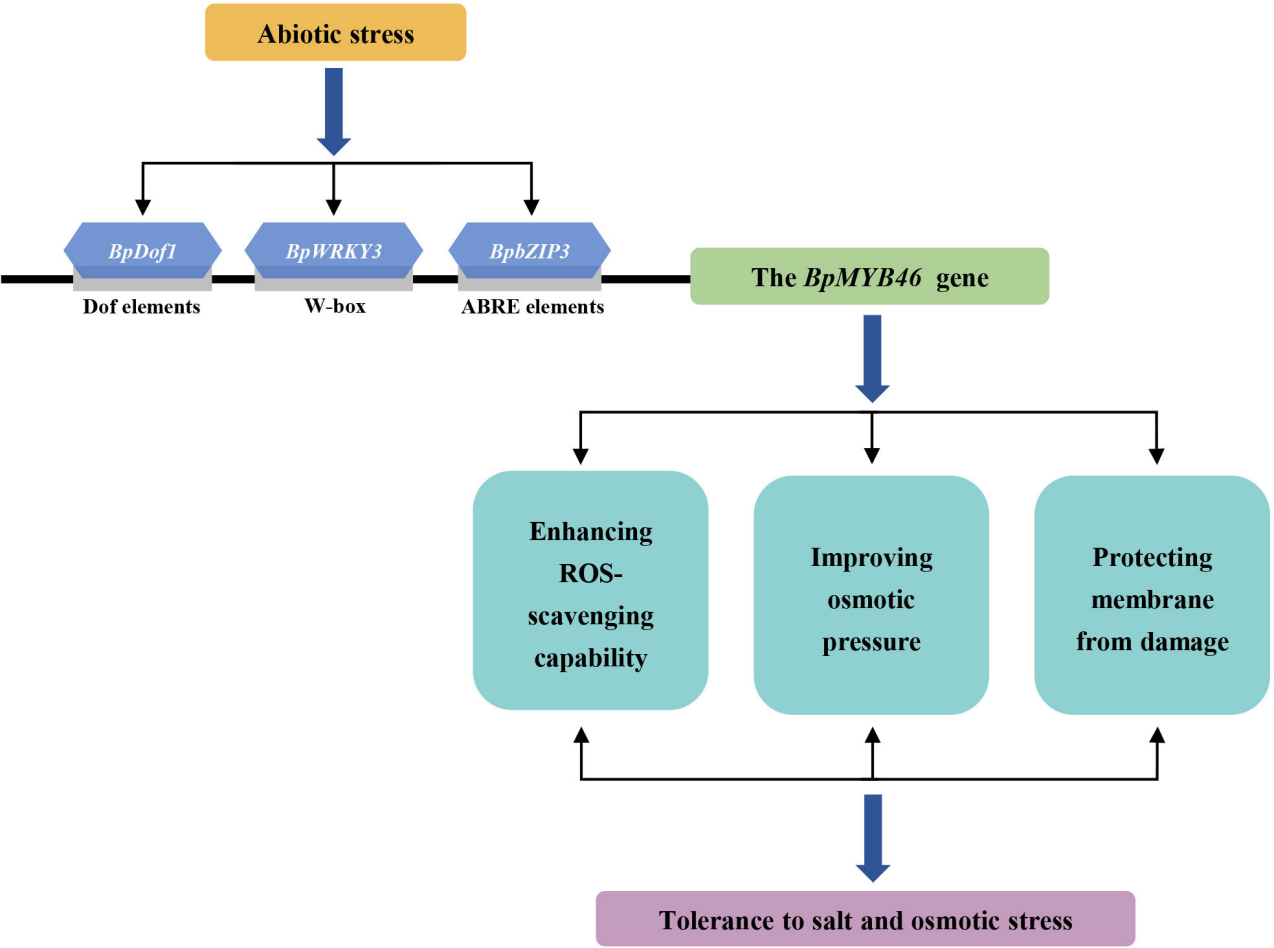
Model of the upstream regulatory network of BplMYB46 involved in abiotic stress responses. Abiotic stress, such as salt or osmotic stress, significantly induce the expression of *BpDof1*, *BpWRKY3*, and *BpdZIP3*. The activated BpDof1, BpWRKY3, and BpdZIP3 then separately binds to Dof, W-box and ABRE elements to regulate the expression of *BplMYB46* gene, which result in significant physiological changes, including reduced reactive oxygen species (ROS) accumulation and membrane damage, and improved osmotic pressure, leading to enhanced salt and osmotic stress tolerance.

Based on the present results, we propose a model describing the upstream regulation of BplMYB46 in the response to abiotic stress (**Figure 13**). Abiotic stress, such as salt or osmotic stress, can induce the expression of *BpDof1*, *BpWRKY3*, and *BpbZIP3*. The activated BpDof1, BpWRKY3, and BpbZIP3 then separately specifically binds to Dof, W-box, and ABRE elements to regulate the expression of *BplMYB46* gene. *BplMYB46* gene significantly altered the expression of its target genes, and triggered physiological changes, including reduced ROS accumulation and membrane damage, improved osmotic pressure, which ultimately enhanced salt and osmotic stress tolerance in birch plant.

## Conclusion

Analyses of GUS staining and activity driven by the *BplMYB46* promoter revealed that the *BplMYB46* promoter exhibits temporal and spatial expression specificity and its expression can be induced by salt and osmotic treatment *in vivo*. Three upstream regulatory factors of BplMYB46, BpDof1, BpWRKY3, and BpbZIP3, were identified using Y1H and ChIP assays. GUS activity and qRT-PCR revealed that BpDof1, BpWRKY3, and BpbZIP3 can regulate the expression of *BplMYB46* by specifically binding to Dof, W-box, and ABRE elements in the *BplMYB46* promoter, respectively. BpDof1, BpWRKY3, and BpbZIP3 were all localized to the nucleus and enhanced tolerance to salt and osmotic stress when they were overexpressed in birch plants. Our study displayed that the upstream regulatory factors of BplMYB46 were identified and their overexpressing birch plants increased the stress tolerance, therefore provided the new candidate genes for breeding of new forest tree varieties with resistance to adverse environments.

## Data availability statement

The datasets presented in this study can be found in online repositories. The names of the repository/repositories and accession number(s) can be found in the article/**Supplementary Material**.

## Author contributions

HG and YW designed the study. XS, BW, and HS provided reagents and materials for the experiments. XS, BW, DW, and HS participated in the experiments, and analyzed the data. HG and YW drafted the manuscript. All authors read and approved the final manuscript.

## Funding

This study was supported by the National Natural Science Foundation of China (31700587) and the Xingliao Talents Program (XLYC1902007).

## Conflict of interest

The authors declare that the research was conducted in the absence of any commercial or financial relationships that could be construed as a potential conflict of interest.

## Publisher’s note

All claims expressed in this article are solely those of the authors and do not necessarily represent those of their affiliated organizations, or those of the publisher, the editors and the reviewers. Any product that may be evaluated in this article, or claim that may be made by its manufacturer, is not guaranteed or endorsed by the publisher.
