# Supplementary material for: The upstream regulatory mechanism of BplMYB46 and the function of upstream regulatory factors that mediate resistance to stress in Betula platyphylla
Source: Front Plant Sci. 2022 Oct 25;13:1030459. doi: 10.3389/fpls.2022.1030459 (PMC9640943; doi:10.3389/fpls.2022.1030459)
Supplement: Supplementary file 1 [file DataSheet_1.docx]

>BpWRKY3[complete cds]

ATGGAGAAGAAGGAGGCGATAAAGACGGAGGAATATCAGACCGTTGGATC

TTCATCGTTCTCGGATCATATATCAGACGGTTATCCATTTTCGGGCATAT

TCGATTTCTCTGAAGGTGAAAAGAGCTCGCTAGGGTTTATGGAGTTACTG

GGTGTTCAAGCCGACTACAGTAGTAGTCCTTCTCTCTTTGATTTGCCATC

GGCTCCACCATCTGTGGCTCCCTCATCGGCTCCACTTCCGGCTTCAGCCG

TTAAAGACTGTTCTGAGGTTTTGAATCCGCCTGCAACACCCAACTCTTCC

TCTATTTCTTCTGCATCGAGTGAGGCTCTCAATGATCATGATGAACTGAC

TAATAAAGCTGTTGACGACGAAGAAGAGCAACAACAAAAGACCAAGAAAG

TGTTGAAAGCCAAGAAGACAAATCAGAAGAGACAGAGAGAGCCAAGATTC

GCGTTCATGACAAAGAGCGAGGTTGATCATCTGGAAGATGGGTACAGGTG

GAGAAAGTACGGACAAAAAGCTGTGAAGAACAGCCCCTTTCCTAGGAGTT

ACTATCGTTGCACTACTACCTCATGTAATGTGAAGAAACGTGTGGAGCGA

TCTTTCACAGATCCAAGCATTGTTGTAACCACCTACGAAGGGCAACACAT

CCATCCTAGTCCAGTCATGCCTCGCCAGATTCTCGCCGGAGCTCCACCGG

ACTCGGGCTTCTCTGGCGGACGTGCTGCCAGCGGCTTTGCTATGCCAATA

CAAAGAAGCATATGCCATTATCAACAACAACAGCGCCAACCGATATCCCA

TATCCATGCCTTGTCACCTTCGAATTTCGCTAATTATAATGGGTTAACGA

ATAATCCAGCTGGTAATTATCTTCGTGAGATTCGGTTTTGCAACACGGGC

TCTGCTTTGCTCAGAGACCATGGGCTTCTTCAAGACATTGTTCCCTCACA

TATGCTCAAGGAAGAATAG

>BpbZIP3[complete cds]

ATGGCACAATTACCACCCAAGGTGCCAAGCATGACTCAGAATTGGCATTC

TTCCTACCAAAGAATGCCAATTATGGCAAACTTCATTTCCACCACCAACA

ACAATACTACAAACAGTGCAGCTGCAACCGCCATCGCCCAACAACAACAA

CCCTCCTGGGTGGATGAGTTTCTCGACTTCTCATCCGCCAGGCGCGGCGC

CCATAGGCGGTCCATGAGCGACTCCGTCGCCTTCCTCGAGGCTCCGGCCT

TCATCGACGAATGCCGTAACTCCACCACAGCGATGATGCATGGGACCAAC

GCCTTCGACCGATTGGACGACGAGCAGCTCATGTCAATGTTCTCCGACGA

CGTCTCCGCCGTCCTTCCGCCTCCCACGGTATCTTCTTCCAACCCTTCCA

CGCCGTCGGACCACAACAGCATTGACGATGAGAATAAGCCAATAATGGCT

GCGATGATGCATGGGACCAACGCCTTCGACCGATTGGACGACGAGCAGCT

CATGTCAATGTTCTCCGACGACGTCTCCGCCGTCCTTCCGCCTCCCACGG

TATCTTCTTCCAACCCTTCCACGCCGTCGGACCACAACAGCATTGACGAT

GAGAATAAGCCAATAATGGCTGGTCTAGACCATCAGCCCAAAAATGAACC

AGGAGAAGTGGAAAGCTCATGCAACCCTGACCCACATGCGCCTCCGCCCT

CAACCACCCCCTCCGGCGACTCCCCCGTCGATCCTAAGAGGGTTAAAAGA

ATTTTGGCAAACAGGCAATCGGCGCAAAGGTCAAGAGTGAGGAAGCTGCA

GTATATTTCGGAGCTTGAACGGAGTGTGACGTCATTACAGACGGAGGTAT

CAGCATTGTCACCAAGGGTTGCATTCTTGGACCATCAAAGGTTGATTCTT

AATGTTGATAATGGCGCTCTCAAGCAACGCATCGCTGCTTTGGCTCAAGA

TAAACTCTTCAAAGATGCACATCAAGAAGCATTAAAGAAGGAAATAGAGA

GATTAAGGCAAATCTATCACCAACAAAATCTCAAGAAGATGAGCAACCAA

AATAATGCACCATCACAACCACAACCACAACCACAACCACAGCCACAGCA

ACAGCCAACCTCTGATCATCATCATCATCATCATCCCTTGCGATGTACGG

ACAAGGAGCACCTTCTCAACTGA
