# Supplementary material for: The upstream regulatory mechanism of BplMYB46 and the function of upstream regulatory factors that mediate resistance to stress in Betula platyphylla
Source: Front Plant Sci. 2022 Oct 25;13:1030459. doi: 10.3389/fpls.2022.1030459 (PMC9640943; doi:10.3389/fpls.2022.1030459)
Supplement: Supplementary file 2 [file DataSheet_2.docx]

Supplementary Material

# Supplementary Tables

**Table S1. Primer sequences used in constructing p1301-BplMYB46 promoter-GUS**

| **Primer** | **Sequence (5’-3’)** |
| --- | --- |
| p1301-GUS-F | CTACATCTAGATATGTGTGGCTTCAATTGC |
| p1301-GUS-R | CTAGACCATGGAGGCCTCCGGCTTCCTCAT |

**Table S2. Primers used to screen upstream regulator of BplMYB46 for constructing the reporter vector**

| **Primer** | **Sequence (5’-3’)** |
| --- | --- |
| promoter-phis2-F | CTAGGAATTCTATGTGTGGCTTCAATTGCAC |
| promoter-phis2-R | AGCTGAGCTCAAACGGTTTGGAGGGGGACC |
| pHIS2-F | GCCTTCGTTTATCTTGCCTGCTC |
| pHIS2R | CGATCGGTGCGGGCCTCTTC |

**Table S3. Primers used to identify upstream regulator of BplMYB46**

| **Primer** | **Sequence (5’-3’)** |
| --- | --- |
| AD-rec2-F | CTATTCGATGATGAAGATACC |
| AD-rec2-R | TTTTTCAGTATCTACGATT |

**Table S4. Primers used for Y1H**

| **Primer** | **Sequence (5’-3’)** | **Amplicon size (bp)** |
| --- | --- | --- |
| Dof-P+-F | CTAGGAATTCTCAGCGAAAAACTGATTAGCT | 231 |
| Dof-P+-R | AGCTGAGCTCCCACATCATATACACTTTAAT |  |
| nDof-P-F | CTAGGAATTCGAGGTTTTTCCAACATCTTT | 125 |
| nDof-P-R | AGCTGAGCTCAATTTGGATCAATTGGTAG |  |
| pHIS2-DOF-F | AATTCAAAGAAAGAAAGGAGCT | 12 |
| pHIS2-DOF-R | CCTTTCTTTCTTTG |  |
| pHIS2-3mDOF-F | AATTCCCCACCCACCCAGAGCT | 12 |
| pHIS2-3mDOF-R | CTGGGTGGGTGGGG |  |
| W-box-P+-F | CTAGGAATTC ATTGTAATTTGACTAATCAT | 199 |
| W-box-P+-R | AGCTGAGCTCAAACGTAAACCATTTTATGC |  |
| nW-box-P-F | CTAGGAATTCTCTTTTTCTAAATCATTTTTG | 157 |
| nW-box-P-R | AGCTGAGCTCAAACGTAAACCATTTTATGC |  |
| W-box-F | AATTCCTGACTCTGACTCTGACTGAGCT | 18 |
| W-box-R | CAGTCAGAGTCAGAGTCAGG |  |
| mW-box-F | AATTCACACACACACACACACACGAGCT | 18 |
| mW-box-R | CGTGTGTGTGTGTGTGTGTG |  |
| ABRE-P+-F | CTAGGAATTCCACGTGCACAAATATTATCAT | 187 |
| ABRE-P+-R | AGCTGAGCTCCATGCTACTCATCACAAAAAAT |  |
| nABRE-P-F | CTAGGAATTCTGGCTTGCATGAATGAGAG | 133 |
| nABRE-P-R | AGCTGAGCTCCATGCTACTCATCACAAAAAAT |  |
| ABRE-F | AATTCCACGTGCACGTGCACGTGGAGCT | 18 |
| ABRE-R | CCACGTGCACGTGCACGTGG |  |
| mABRE-F | AATTCACAACAACAACAACAACAGAGCT | 18 |
| mABRE-R | CTGTTGTTGTTGTTGTTGTG |  |

**Table S5. Primer sequences used for ChIP assay**

| **Primer** | **Sequence (5’-3’)** | **Amplicon size (bp)** |
| --- | --- | --- |
| Dof1-GFP-F | CACGGGGGACTCTAGAATGATCCAAGAACTGTTGGG | 933 |
| Dof1-GFP-R | TGCTCACCATACTAGTAGGATATGCACCATTAGTAG |  |
| bZIP3-GFP-F | CACGGGGGACTCTAGAATGGCACAAT TACCACCCAAG | 1173 |
| bZIP3-GFP-R | TGCTCACCATACTAGTGTTGAGAAGGTGCTCCTTGT |  |
| WRKY3-GFP-F | CACGGGGGACTCTAGAATGGAGAAGAAGGAGGCGAT | 969 |
| WRKY3-GFP-R | TGCTCACCATACTAGTTTCTTCCTTGAGCATATGTG |  |
| Dof-ChIP-F | AGGCAGAAAGAAAGGAGGTT | 145 |
| Dof-ChIP-R | CACTTTAATTTGGATCAATTG |  |
| W-box-ChIP-F | ATTGTAATTTGACTAATCAT | 140 |
| W-box-ChIP-R | CAAGTTGACCAATTCTATAC |  |
| ABRE-ChIP-F | CCACGTGCACAAATATTATC | 146 |
| ABRE-ChIP-R | TTCTAACCAAGCCAAGGTAT |  |
| No-motif-ChIP-F | TTAGTCTTATCTTAAGAGTG | 140 |
| No-motif-ChIP-R | ATTACAATTACATTTCACT |  |
| a-tubulin-F | TGGCTCGAATGCACTGTTGG | 215 |
| a-tubulin-R | TCAACCGCCTTGTCTCTCAGG |  |

**Table S6. Primers of transcription factors used for transient transformation**

| **Primer** | **Sequence (5’-3’)** | **Amplicon size (bp)** |
| --- | --- | --- |
| Dof-P+-F-GUS | CTAGAAGCTTTCAGCGAAAAACTGATTAGCT | 278 |
| Dof-P+-R-GUS | CATGCCATGGCCGTGTTCTCTCCAAATGAAATGAACTTC  CTTATATAGAGGAAGGGTCCACATCATATACACTTTAAT |  |
| nDof-P-F-GUS | CTAGAAGCTTGAGGTTTTTCCAACATCTTT | 172 |
| nDof-P-R-GUS | CATGCCATGGCCGTGTTCTCTCCAAATGAAATGAACTT  CCTTATATAGAGGAAGGGTAATTTGGATCAATTGGTAG |  |
| W-box-P+-F-GUS | CTAGAAGCTTATTGTAATTTGACTAATCAT | 246 |
| W-box-P+-R-GUS | CATGCCATGGCCGTGTTCTCTCCAAATGAAATGAACTTC  CTTATATAGAGGAAGGGTAAACGTAAACCATTTTATGC |  |
| nW-box-P-F-GUS | CTAGAAGCTTTCTTTTTCTAAATCATTTTTG | 204 |
| nW-box-P-R-GUS | CATGCCATGGCCGTGTTCTCTCCAAATGAAATGAACTT  CCTTATATAGAGGAAGGGTAAACGTAAACCATTTTATGC |  |
| ABRE-P+-F-GUS | CTAGAAGCTTCACGTGCACAAATATTATCAT | 234 |
| ABRE-P+-R-GUS | CATGCCATGGCCGTGTTCTCTCCAAATGAAATGAACTTC  CTTATATAGAGGAAGGGTCATGCTACTCATCACAAAAAAT |  |
| nABRE-P-F-GUS | CTAGAAGCTTTGGCTTGCATGAATGAGAG | 180 |
| nABRE-P-R-GUS | CATGCCATGGCCGTGTTCTCTCCAAATGAAATGAACTTCC  TTATATAGAGGAAGGGTCATGCTACTCATCACAAAAAAT |  |
| Dof1-prokII-F | CTCTAGAGGATCCCCATGATCCAAGAACTGTTGGG | 933 |
| Dof1-prokII-R | TCGAGCTCGGTACCCTCAAGGATATGCACCATTAG |  |
| WRKY3-prokII-F | CTCTAGAGGATCCCCATGGAGAAGAAGGAGGCGAT | 969 |
| WRKY3-prokII-R | TCGAGCTCGGTACCCCTATTCTTCCTTGAGCATAT |  |
| bZIP3-prokII-F | CTCTAGAGGATCCCCATGGCACAATTACCACCCAAG | 1173 |
| bZIP3-prokII-R | TCGAGCTCGGTACCCTCAGTTGAGAAGGTGCTCCT |  |

**Table S7. Primers of BplMYB46 used for qRT-PCR**

| **Primer** | **Sequence (5’-3’)** | **Amplicon size (bp)** |
| --- | --- | --- |
| Ubiquitin-F | GGAGGACAAGGTGGAGGG | 228 |
| Ubiquitin-R | GATTGAGGGGAGGGATGC |  |
| a-tubulin-F | TGGCTCGAATGCACTGTTGG | 215 |
| a-tubulin-R | TCAACCGCCTTGTCTCTCAGG |  |
| *MYB-s* | TCAGGTGGAGGTGAGAAA | 271 |
| *MYB-r* | CAAGAAGGGAGTGCAAAT |  |

**Table S8. Primer sequences used in the analysis of transcription factors for qRT-PCR**

| **Primer** | **Sequence (5’-3’)** | **Amplicon size (bp)** |
| --- | --- | --- |
| Ubiquitin-F | GGAGGACAAGGTGGAGGG | 228 |
| Ubiquitin-R | GATTGAGGGGAGGGATGC |  |
| a-tubulin-F | TGGCTCGAATGCACTGTTGG | 215 |
| a-tubulin-R | TCAACCGCCTTGTCTCTCAGG |  |
| Dof1-G-F | TCCTCAACAACAACCTCGTC | 153 |
| Dof1-G-R | GCCTTTCGTCCAATACCG |  |
| WRKY3-G-F | GGCGATAAAGACGGAGGA | 189 |
| WRKY3-G-R | GCCGATGGCAAATCAAAG |  |
| bZIP3-G-F | AACGCATCGCTGCTTTGG | 184 |
| bZIP3-G-R | GTTGGCTGTTGCTGTGGC |  |
